# Striatal dysfunction during reversal learning in unmedicated schizophrenia patients^☆^

**DOI:** 10.1016/j.neuroimage.2013.11.034

**Published:** 2014-04-01

**Authors:** Florian Schlagenhauf, Quentin J.M. Huys, Lorenz Deserno, Michael A. Rapp, Anne Beck, Hans-Joachim Heinze, Ray Dolan, Andreas Heinz

**Affiliations:** a Department of Psychiatry and Psychotherapy, Campus Charité Mitte, Charité — Universitätsmedizin Berlin, Germany; b Max Planck Institute for Human Cognitive and Brain Sciences, Leipzig, Germany; c Gatsby Computational Neuroscience Unit and Wellcome Trust Centre for Neuroimaging, University College London, London WC1N 3BG, UK; d Translational Neuromodeling Unit, Department of Biomedical Engineering, ETH Zurich and University of Zurich, Switzerland; e Department of Psychiatry, Psychotherapy and Psychosomatics, University Hospital of Psychiatry Zurich, Zurich, Switzerland; f Department of Psychiatry, Mount Sinai School of Medicine, New York, NY, USA; g Leibniz Institute for Neurobiology, Otto-von-Guericke University, Magdeburg, Germany; h Department of Neurology, Otto-von-Guericke University, Magdeburg, Germany; i Wellcome Trust Centre for Neuroimaging, University College London, London WC1N 3BG, UK; j Humboldt-Universität zu Berlin School of Mind and Brain, Berlin, Germany; k Cluster of Excellence NeuroCure, Charité-Universitätsmedizin Berlin, Berlin, Germany

**Keywords:** Schizophrenia, Reversal learning, Imaging, Reward, Ventral striatum, Computational modeling

## Abstract

Subjects with schizophrenia are impaired at reinforcement-driven reversal learning from as early as their first episode. The neurobiological basis of this deficit is unknown. We obtained behavioral and fMRI data in 24 unmedicated, primarily first episode, schizophrenia patients and 24 age-, IQ- and gender-matched healthy controls during a reversal learning task. We supplemented our fMRI analysis, focusing on learning from prediction errors, with detailed computational modeling to probe task solving strategy including an ability to deploy an internal goal directed model of the task. Patients displayed reduced functional activation in the ventral striatum (VS) elicited by prediction errors. However, modeling task performance revealed that a subgroup did not adjust their behavior according to an accurate internal model of the task structure, and these were also the more severely psychotic patients. In patients who could adapt their behavior, as well as in controls, task solving was best described by cognitive strategies according to a Hidden Markov Model. When we compared patients and controls who acted according to this strategy, patients still displayed a significant reduction in VS activation elicited by informative errors that precede salient changes of behavior (reversals). Thus, our study shows that VS dysfunction in schizophrenia patients during reward-related reversal learning remains a core deficit even when controlling for task solving strategies. This result highlights VS dysfunction is tightly linked to a reward-related reversal learning deficit in early, unmedicated schizophrenia patients.

## Highlights

• Probabilistic reversal learning was examined in unmedicated schizophrenia patients.
• Computational modeling assessed subjects ability to use the latent task structure.
• SZ patients showed lower reinforcement sensitivity and higher switch tendency.
• Blunted striatal prediction error signal in unmedicated schizophrenia patients.
• PFC activation during reversal errors intact in SZ patients able to do the task.

## Introduction

Schizophrenia is characterized by severe and characteristic dysfunctions of affect, thought (inference) and behavior (Andreasen, 1999). Tasks that use rewards as feedback and require the inference of correct and changing response strategies tap into each of these three domains. Schizophrenia impairs performance on tasks sensitive to these dimensions, including the Wisconsin Card Sorting Task or reversal learning. In the latter, subjects have to choose between possibilities (e.g. pressing a left or right button) where choice is probabilistically rewarded (e.g. the right button in 80% and the left in 20%), thus eliciting errors in reward prediction. Moreover, unannounced reversals of the “better” choice demands flexible behavioral adjustment (e.g. a switch to the previously less rewarded left side) (Elliott et al., 1995; Hutton et al., 1998; Pantelis et al., 1997). Recent work on reversal learning has yielded three key findings.

First, deficits are present in first episode psychosis (Leeson et al., 2009; Murray et al., 2008a; Waltz and Gold, 2007) which argues against deficits as a secondary consequence of long-term medication, nor are they a late feature of a chronic schizophrenic state (Elliott et al., 1995; Pantelis et al., 1997). Moreover, a recent prospective study of 262 first episode psychosis patients over six years showed that these deficits were stable over time and that reversal learning deficits differentiated between control and patient populations even after controlling for IQ, while other differences did not (Leeson et al., 2009).

Second, recent work has improved our understanding of the neural structures involved in reversal learning in healthy controls. These include the ventral striatum (Cools et al., 2002; Hampton and O'Doherty, 2007; Swainson et al., 2000), the medial PFC (Cools et al., 2002; Hampton and O'Doherty, 2007; Hampton et al., 2006) and the ventral parts of the PFC (Dias et al., 1996; Fellows and Farah, 2003; Hornak et al., 2004) including the lateral orbitofrontal cortex (Chamberlain et al., 2008; Remijnse et al., 2006). The prefrontal areas have been related to cognitively driven, goal-directed (or so called ‘model-based’) reversal learning, while the ventral striatum is implicated in aspects of reversal learning driven by reward prediction errors (PE) to guide behavior (so-called ‘model-free’ learning) (Daw et al., 2005; Hampton et al., 2006; Hare et al., 2008). Since Schultz et al. (Schultz, 2007) suggested that increases in phasic dopamine firing reflect errors of reward prediction, stress-induced or chaotic dopamine firing in acute schizophrenia (Heinz, 2002; Kapur, 2003) might impair PE signaling in the ventral striatum and hence ‘model-free’ learning from reinforcement.

Third, functional imaging studies in other reward learning tasks have strengthened the hypothesis that dopaminergic dysfunction is associated with reversal learning impairment in schizophrenia. Two findings are key: first, the hyperdopaminergic state of the striatum in schizophrenia (Howes et al., 2012), which may impair reversal learning similarly to the effect of l-DOPA in Parkinson's disease patients (Cools et al., 2001, 2007); and second, striatal hypoactivation is seen during reward processing in schizophrenia (Juckel et al., 2006b; Koch et al., 2010; Murray et al., 2008b; Schlagenhauf et al., 2009; Waltz et al., 2009).

However, previous imaging studies have not studied early psychosis, nor medication free patients resulting in a significant pharmacological drug confound (Juckel et al., 2006a; Nielsen et al., 2012a; Schlagenhauf et al., 2008). A further potential confound is the likelihood that disease-related deficits can lead to subjects adopting different strategies in solving tasks (Price and Friston, 1999; Weickert et al., 2010). This is particularly important given the long-standing ‘reality distortions’ even in early psychosis. Imaging differences in these instances might then reflect secondary strategic (cognitive) differences, rather than any more basic disease processes.

Here, we used fMRI to investigate prefrontal and ventral striatal activity during reversal learning in a sample of unmedicated, largely first-episode patients with schizophrenia. We also address the more problematic issue of altered strategy by using detailed computational modeling, and matching patients and controls closely in their ability to infer and use the latent structure of the task. This allows more definitive conclusions to be drawn about processes more directly related to the disease that diminishes problems of interpretation due to behavioral differences associated with adaptive disease dependent strategies.

## Methods and materials

### Participants

24 unmedicated schizophrenia patients and 24 healthy controls matched for age, sex, handedness (Oldfield, 1971) and verbal IQ (Schmidt and Metzler, 1992) completed the study (Table 1). The schizophrenia patients (n = 15 first episode patients) fulfilled DSM-IV and ICD-10 criteria and had no other psychiatric axis I disorder (SCID interviews). The 15 first episode patients were drug-naïve; the remaining 9 received antipsychotic treatment with second generation antipsychotics in the past and had stopped for various reasons for at least three weeks. All patients were recruited during their first days of treatment when they were informed about treatment options and considered medication and psychotherapy.

Outpatients were recruited from the Early Recognition Center of Psychosis and inpatients from the Department of Psychiatry and Psychotherapy, Charité-Universitätsmedizin Berlin (Campus Charité Mitte) (see Table 1 for clinical description). Psychopathological symptoms were assessed with the Positive and Negative Syndrome Scale (PANSS) (Kay et al., 1987). Healthy volunteers had no axis I or II psychiatric disorder (SCID interviews) and no family history of psychiatric disorders in first degree relatives. The local ethics committee approved the study. Written, fully informed consent was obtained from all participants.

### Task

**Fig. 1:**
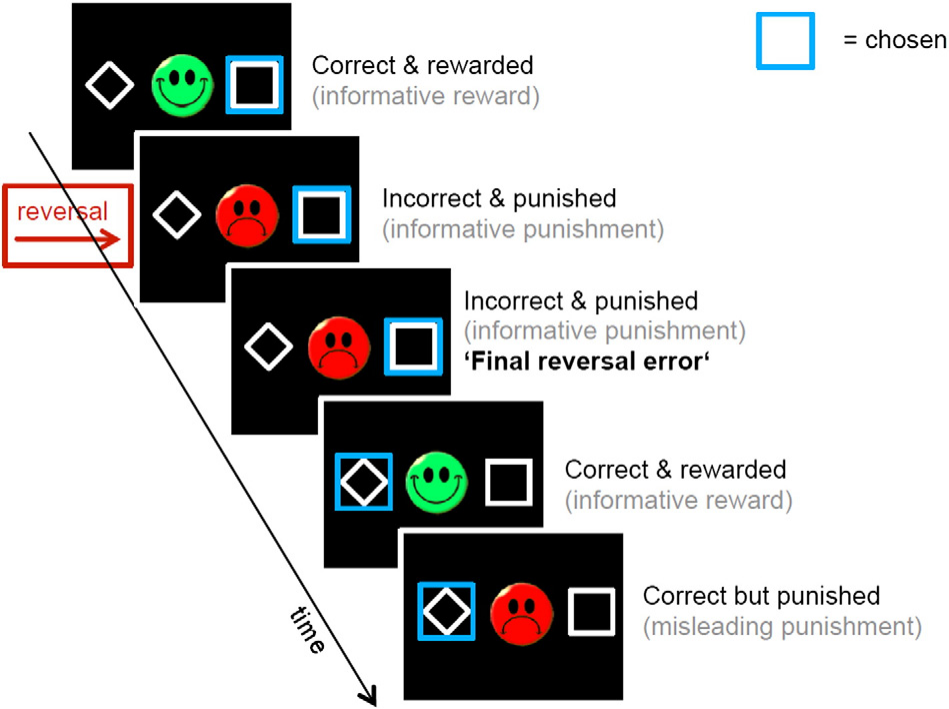
Structure of the reversal learning task. Subjects chose one of two abstract stimuli as quickly as possible by pressing the left or right button (maximum decision time: 2 s). Then, a blue box surrounding their chosen target and feedback (either a green smiley face for positive feedback or a red frowny face for negative feedback) was simultaneously shown for 1 s. “Correct” here indicates choice of the more often rewarded (80%) and “incorrect” of the less rewarded stimulus (20%) during that block. A misleading trial is a trial on which a probabilistic punishment was received although the correct (e.g. more rewarded) stimulus was chosen (shown), or one on which a probabilistic reward was obtained despite the incorrect choice (not shown). Note that subjects did not know whether feedbacks were truly informative or not, as they did not know the underlying state of the task. Participants were only able to label feedback as informative or misleading based on their own beliefs about the state of the task, i.e. about which response is more rewarded.

During fMRI acquisition, participants performed two sessions of 100 trials of a reversal learning task (Fig. 1) with three types of blocks (Kahnt et al., 2009; Park et al., 2010; Schlagenhauf et al., 2012). In block type 1, a reward was delivered for choosing the stimulus on the right-hand side if less than 80% of the recent right-hand choices had been rewarded, and a punishment delivered otherwise. Conversely, a punishment was delivered for choosing the left-hand stimulus if less than 80% of the recent left-hand choices had been punished, and a reward delivered otherwise. In block type 2, the contingencies were simply reversed for the left and right side. In block type 3, the probabilities were 50/50 instead of 80/20. Switches between blocks happened after 16 trials, or at any time after 10 trials once subjects reached 70% correct choices. The trials were separated with a jittered interval of 1–6.5 s. Before entering the scanner, subjects performed a practice version of the task (without reversal component) so as to be familiarized with the probabilistic character of the task.

### Behavioral data analyses

Classical statistical tests were performed using SPSS Version 18. Number of correct responses and achieved reversal stages were compared using two-sample t-tests. Behavioral adaptation was assessed using a 2 × 10 repeated measures ANOVA with group as between-subject factor and trial (first ten trials after reversal occurred) as within-subjects factor. Greenhouse–Geiser correction was applied when necessary.

### Computational modeling of behavior

Three computational models were implemented in Matlab (Mathworks, v7.5-7.10) and fitted to the data:

1) Standard Rescorla–Wagner models (stimulus-action, SA), where the Q-value of only the chosen option was updated by a prediction error. This model explains the observed behavior by computing for each trial t an action value *Q_t_* (*a_t_*) which represents the expected outcome of the action *a_t_*. The Q-value of the chosen action is updated iteratively by a prediction error which denotes the difference between the expected outcome *Q*_*t* − 1_ (*a_t_*) and the actual received outcome *R*. The influence of the prediction error is scaled by the learning rate *ε*. This results in the following equation:
  $$Q_{t}\left(a_{t}\right)=Q_{t-1}\left(a_{t}\right)+\varepsilon \left(R-Q_{t-1}\left(a_{t}\right)\right).$$
2) Double update models (DSA), where the Q values for both actions were updated on every trial. This model used the same Q-learning algorithm as the standard Rescorla–Wagner model with the important difference that the *Q* values for both the chosen and the unchosen actions are updated on every trial. If a reward is obtained for action *a*, the *Q*-value for this action is increased, while the *Q*-value of the other, unchosen action $\bar{a}$ is reduced. This model takes the structure of the task into account and represents the asymmetry of the two actions of the reversal learning task: If action *a* is the good option (rewarded with 80%), then the other action $\bar{a}$ is the bad option (rewarded with 20%).
3) Hidden Markov Models (HMM), which suppose that participants choose their action based on their belief about the underlying state of the task. This model implies that subjects were able to recognize the existence of a latent state, draw inferences about it, and use these to guide behavior. The HMM assumes that subjects use the past history of choices and resulting rewards to maintain a belief about the current states of the task. The belief about the current state is used to make a choice. Unlike the Q-learning algorithms the HMM builds a model of the task state by inferring a belief about the current state e.g. the good stimulus is on the left-hand side (state 1) or the good stimulus is on the right-hand side (state 2). Based in the current belief about the state and based on a change in terms of what the next belief is, the HMM can be used to differentiate different trial types: informative rewards (consistent choice rewarded and resulting in belief stay or inconsistent choice rewarded and resulting in belief switch), probabilistic punishments (consistent choice punished but resulting in belief stay or inconsistent choice punished but resulting in belief switch), informative punishments (consistent choice punished and resulting in belief switch and inconsistent choice punished and resulting in belief stay), and probabilistic rewards (consistent choice rewarded but resulting in belief switch or inconsistent choice rewarded but resulting in belief stay). ‘Staying’ and ‘switching’ were defined in terms of what the next belief was, not what the next action was.

We tested two versions of each model, in which rewards and punishments had either equal or differing (below denoted as R/P = reward/punishment) effects. Model comparison was performed by computing Bayes factors at the group level (see Suppl. Material). We used this random-effects Bayesian inference technique (see Suppl. Material) to infer each subject's parameters in order to generate regressors that closely match that particular persons' behavior. If the same parameters would be used for every subject the individual behavior would be more or less explained by those parameters and this would then be expressed as differences in the correlation between the regressor and the BOLD signal. This can be minimized by fitting parameters to the observed individual's behavior and use those parameters to generate regressors that closely match that particular persons' behavior. In our analyses, we therefore used regressors fitted to each subject individually in order to ‘control’ for alterations in that learning process (due to changes in parameters).

### FMRI

#### fMRI acquisition

Imaging was performed using a 3 Tesla GE Signa scanner with a T2*-weighted sequence (29 slices with 4 mm thickness, TR = 2.3 s, TE = 27 ms, flip = 90°, matrix size = 128 × 128, FOV = 256 × 256 mm^2^, in-plane voxel resolution of 2 × 2 mm^2^) and a T1-weighted structural scan (TR = 7.8 ms, TE = 3.2 ms, matrix size 256 × 256, 1 mm slice thickness, voxel size of 1 mm^3^, flip = 20°).

#### fMRI data analyses

Functional imaging data was analyzed using SPM8 (http://www.fil.ion.ucl.ac.uk/spm/software/spm8/). Preprocessing included correction for delay for slice time acquisition and for motion, spatial normalization into Montreal Neurological Institute (MNI) space and spatial smoothing with 8 mm FWHM-kernel (see Suppl. Material for further details). One patient's imaging data was corrupted and discarded.

##### Neural correlates of prediction errors

In a first step and regardless of differences in task strategy, BOLD prediction error correlations were analyzed as in various previous published studies (e.g. Daw et al., 2006; O'Doherty et al., 2004; Park et al., 2010). The images were analyzed in an event related manner using the general linear model approach (GLM) as implemented in SPM8. Neuronal activity was modeled for win and loss trials separately by stick functions at the onset of feedback. We used a parametric design (Buchel et al., 1998), in which the trial-by-trial PE values from the Rescorla–Wagner model (SA) modulated the amplitude of the trial related stick. Regressors of interest for the BOLD-responses, corresponding to the trial-wise PEs, were generated by convolving the modulated stimulus functions with the canonical hemodynamic response function (HRF), provided by SPM8. To account for signal fluctuations associated to movement by susceptibility interaction, the six movement parameters from the realignment pre-processing step were included in the model as additional regressors. The individual contrast images for the contrast of the PE modulated feedback (combining win and loss feedback) were then taken to a random effects group-level analysis using one- and two-sample t-tests. One schizophrenia patient was an outlier with regard to the bilateral VS PE signal (z-value = 3.1) and was therefore excluded from further analyses. For correction of multiple comparisons family wise error (FWE) correction was applied using small volume correction within the VS. The VS VOI was constructed based on coordinates of previous findings as in (Schlagenhauf et al., 2012) using an in house tool to create an fMRI-literature based probabilistic VOI for the VS (see Suppl. Material for more details). Activation outside the VS VOI is reported at p < 0.05 FWE corrected for the whole brain and group comparisons outside the ventral striatum were restricted to areas showing a task main effect for both groups taken together at p_FWE whole brain corrected_ < 0.05. Additionally, we report results significant at p < 0.05 FDR corrected at the cluster level with an initial inclusion threshold of p < 0.01 uncorrected (see Supplementary Results Table S1–S2).

##### Neural correlates of subjectively perceived reversal errors

In order to assess neuronal activation during behavioral adaptation, we examined behaviorally salient trials identified via the HMM model (empirical reason for model selection see below). Event types were defined in terms of subjective task states estimates as inferred by the HMM model rather than in terms of true task states, which was only available to the experimenter. This allows analyzing errors, subjectively inferred, as being informative for behavioral adaptation.

The HMM R/P model was used to classify reward and punishment trials according to the subjects' belief about their informativeness. Four regressors were defined at the single subject level: 1) rewards and 2) punishments judged as informative by the subjects, and 3) rewards or 4) punishment judged as not informative by the subjects. An informative reward was defined as one that occurred after a choice that was consistent with the participant's belief about the state of the experiment, and that was followed by a ‘stay’ in belief state. A reward was also an informative reward if the model indicated, for instance, that subjects believed the left side to be correct, but erroneously chose the right, still received a reward and then switched to the right. Similarly, an informative punishment was one that occurred after a choice consistent with the subjects' belief and followed by a switch in their belief about the current state of the task. This thus defines informative events as seen by the subject, rather than as imposed by the experimenter. For example a trial was labeled as ‘informative reward’ either if the subject made a choice compatible to the belief state which choice would be better (believed to be in state s1 where action a is better and made action a), got a reward and the belief about the state stayed the same or if the subject made a choice of the worse option according to his/her belief state (believed to be in state s1 where action a is better but made action $\bar{a}$), got a reward and this led to a switch of the belief state form s1 to s2. ‘Staying’ and ‘switching’ were defined in terms of what the next belief was, not what the next action was. Additional regressors were included for invalid trials and for the six movement parameters.

At the second level (group comparison between schizophrenia patients and controls), contrast ‘informative punishment – informative reward’ was assessed and compared between both groups differentiated for model fit of the HMM R/P (see Suppl. Material and Results 3.3) using a flexible factorial analysis of variance with the factors condition (informative rewards, informative punishments, non-informative punishments), and group (controls vs. patients) treating subjects as a random factor.

Ventral striatal small volume correction was used with an 8 mm sphere centered at x, y, z = [+/− 10, 8, − 4] according to Cools et al. (2002) and is reported at p_FWE-corrected for VS VOI_ < 0.05. Activation outside the VS is reported at p < 0.05 FWE corrected for the whole brain and group comparisons outside the ventral striatum were restricted to areas showing a task main effect for both groups taken together at p_FWE whole brain corrected_ < 0.05. Additionally we report results significant at p < 0.05 FDR corrected at the cluster level with an initial inclusion threshold of p < 0.01 uncorrected (see Supplementary Results Table S3–S4).

Model-based fMRI analyses may be confounded by differences in model fit between the compared groups. This could lead to a less accurate description of the times at which different cognitive processes occurred and could lead to spurious cross-group differences. We addressed this issue by only including subjects whose behavior was described by the model equally well and used a random-effects Bayesian inference technique to infer each subjects' parameters (see Supp. Material). Furthermore, in additional analyses, the individual model fit (predictive probability) was included as covariate into the second level group analyses.

## Results

### Behavioral analysis

Schizophrenia patients (SZ) performed worse than healthy controls (HC), with 64.3 +/− 7.6 vs. 75.0 +/− 6.4% correct responses (t = 5.31, p < 0.001). Mean correct choices after a reversal in a 2 × 10 repeated-measures ANOVA showed a significant main effect of trial (F(3.04,140.15) = 53.144, p < 0.001), a significant main effect of group (F(1,46) = 22.312, p < 0.001) and a significant group by trial interaction (F(3.04, 140.15) = 3.726, p = 0.012). Reversals in SZ were less often triggered by achieving the criterion for reversals (SZ: 6.1 +/− 3.5, HC: 9.9 +/− 3.0, two sample *t*-test t = − 4.04, p < 0.001) and instead happened more often after reaching the maximal trials number (number of reversal blocks SZ: 11.0 +/− 1.6; HC: 12.5 +/− 1.5).

### Neural correlates of prediction errors

**Fig. 2:**
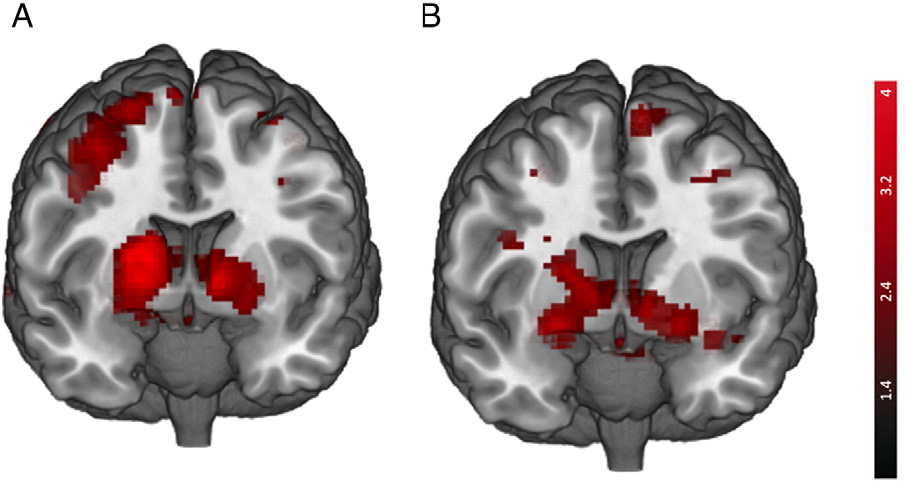
Prediction error signal in the ventral striatum. A: Prediction error signal in 24 healthy controls. B: Stronger PE signal in healthy controls (n = 24) compared to unmedicated schizophrenia patients (n = 22).

Assessing the correlation between the prediction error (PE) derived from the Rescorla–Wagner model and the BOLD signal, the group of 24 healthy controls displayed a significant VS PE signal (R: t = 3.319, [17 3 − 5], p_FWE corrected for VS VOI_ = 0.015; L: t = 3.161, [− 11 8 − 3], p_FWE corrected for VS VOI_ = 0.025) (see Fig. 2A). There was no significant VS activation in the group of 22 schizophrenia patients (p_FWE corrected for VS VOI_ > .2). A contrast of all 24 healthy controls with the 22 patients with schizophrenia revealed a significant bilateral group difference in the VS (R: t = 2.466, [20 3 − 8], p_FWE corrected for VS PE VOI_ = 0.049; L: t = 2.562, [− 11 6 − 5], p_FWE corrected for VS VOI_ = 0.047) (see Fig. 2B). There were no significant group activation or group differences outside the VS VOI when applying FWE whole brain correction (see also Supp Table 1 and 2).

### Computational modeling of behavior

**Fig. 3:**
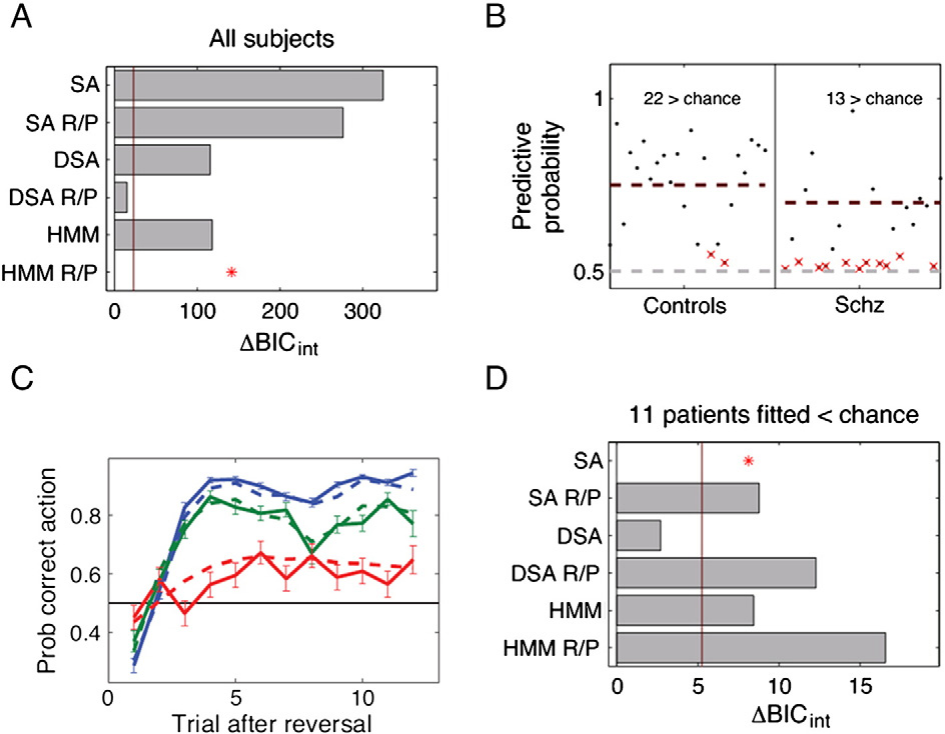
Model comparison. A: Model Bayesian Information Criterion (∆BIC_int_) scores (compared to the best model). The best model has the lowest score (∆BIC_int_ = 0). The red line shows the random effects threshold. B: Model fit to individual participants (black dots). Red crosses indicate participants not fitted better than chance. Red dashed lines show group means for participants fitted better than chance. C: Average learning curves after reversals for participants fitted worse than chance (red), and for HC and SZ fitted better than chance (blue and green, respectively). Dashed lines show action choices generated from the model: after fitting the parameters to each subject's data, the model was run on the same task and surrogate choices were generated. D: Model ∆BIC_int_ scores for poor-fit schizophrenia patients (fitted worse than chance). Asterisks indicate the best fitting model. For further details see Supplementary Results. Abbreviations: SA = stimulus-action; DSA = Double stimulus-action, double update model; HMM = Hidden Markov Model; R/P = models with separate reward and punishment effects.

Bayesian model comparison showed that a Hidden Markov Model provided the most parsimonious account of behavior (Fig. 3A). This model fitted 22/24 control participants, but only 13/24 patients were fitted better than chance (Fig. 3B). Controls and good fitting patients displayed behavioral adaptation after reversals, while poorly fitting patients did not (Fig. 3C).

Repeating behavioral analyses (see Section 3.1) for these three groups using a group (good-fit HC, good-fit SZ, poor-fit SZ) × trial (first ten trials after reversal occurred) repeated-measures ANOVA revealed a significant main effect of trial (F(3.94,173.69) = 7.604, p < 0.001), a significant main effect of group (F(3,44) = 11.697, p < 0.001) and a significant group by trial interaction (F(11.84,173.69) = 5.377, p < 0.001) (Fig. 3C). A one-way ANOVA of the number of achieved reversals (number of blocks in which criteria was reached) showed a significant group effect (F(3,47) = 4.556, p = 0.007) and post-hoc t-tests with Bonferroni-correction revealed that poor-fit SZ patients differed from both other groups (p-values < 0.05), while no group difference was evident between good-fit HC and good-fit SZ (p > 0.8). Examining the 11 poor-fit schizophrenia patients alone (Fig. 3D) revealed that the most parsimonious (though emphatically still poor) model was the most simple, namely a Rescorla–Wagner model (SA), which neglects the reciprocity between the two stimuli and the step-wise switch task structure.

To explore differences in model fit within the patients we applied a step-wise linear regression analysis. This showed that model fit was predicted only by positive symptoms (β = − 0.463, t = − 2.452, p = 0.02) but not by negative symptoms or other PANSS scores, nor by premorbid intelligence or attention (all p-values > 0.3). This indicates that patients with more severe positive symptoms were less able to infer and use the latent structure of the task.

**Fig. 4:**
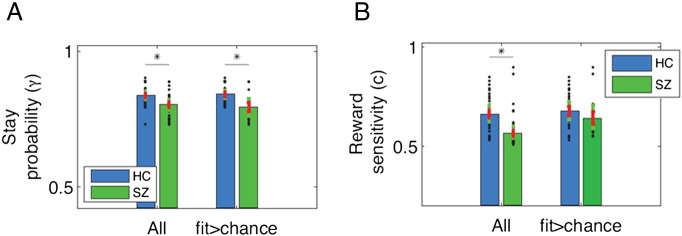
Group differences for HMM parameters. Comparison of model parameters between healthy controls (blue) and schizophrenia patients (green) for stay probability parameter γ (A) and reward sensitivity c (B). Stay probability was significantly different between HC and all SZ (n = 22), and remained so when excluding participants fitted worse than chance. Reward sensitivity, on the other hand, did not differ between HC and SZ who were well fitted, but did indeed differ if the poorly fitted subjects were included.

The HMM contained three parameters: two captured participants' sensitivity to rewards and punishments; a further parameter γ captured their beliefs about how likely the task state was to remain the same on each trial. Controls and schizophrenia patients differed in the model parameters of reward sensitivity and stay probability (MANOVA comparing all HC (n = 24) vs. all SZ (n = 22): F(3,44) = 6.147, p < 0.001). Again, looking at the three subgroups (good-fit HC (n = 22), good-fit SZ (n = 13), and poor-fit SZ (n = 11)) this revealed specific group differences compared to controls (MANOVA HC vs. good-fit SZ vs. poor-fit SZ: F(6,84) = 7.595, p < 0.001). Post-hoc t-tests showed that reward sensitivity differed between controls and poor-fit patients (p < 0.001 Bonferroni corrected, with poor-fit patients displaying reward insensitivity), but not between controls and good-fit patients (p > 0.3 Bonferroni corrected; Fig. 4A). As seen in the learning curves (Fig. 3A), the subgroup of patient who was poorly fitted by the HMM, and who displayed low reward sensitivity, also showed a blunted learning curve indicating little behavioral adaptation. On the other hand, the stay probability was significantly different between controls and poorly fitted as well as between controls and well fitted patients (p < 0.001 and p = 0.003 respectively, Bonferroni corrected; Fig. 4B). Thus, patients' higher tendency to switch differentiated them from controls even after excluding participants who were not sensitive to the task structure.

To obviate a concern that the above observed group difference in ventral striatal prediction error signaling might be due to differences in model fit, we conducted additional analyses using two different approaches. First we repeated the initial RW analysis (see result section: Neural correlates of prediction errors) in the two groups including only subjects with good model fit (22 good-fit HC vs. the 13 good-fit schizophrenia patients). Here group differences approached significance bilaterally in the VS (R: t = 2.370, [20 3 − 8], p_FWE corrected for VS PE VOI_ = 0.068; L: t = 2.428, [− 11 6 − 5], p_FWE corrected for VS PE VOI_ = 0.072). In a second set of analyses we included the individual likelihood (predictive probability) as a covariate. And found no significant correlation between the VS PE signal and the predictive probability neither in a one-sample *t*-test across all patients (n = 22) and controls (n = 24) taken together (p_FWE corrected for VS PE VOI_ > .3) nor in a one-sample *t*-test including only the patients (p_FWE corrected for VS PE VOI_ > .5). Furthermore, when including the predictive probability in the initial group comparison (2-sample *t*-test comparing 24 HC vs. 22 SZ), the group differences in the VS remained significant (R: t = 2.553, [20 3 − 8], p_FWE corrected for VS PE VOI_ = 0.050; L: t = 3.187, [− 11 6 − 5], p_FWE corrected for VS PE VOI_ = 0.016).

### Neural correlates of subjectivly perceived reversal errors

We finally used the Hidden Markov model (HMM) to approximate participants' beliefs about reversals, rather than the experimenters' objective knowledge about task state (see Methods and Suppl. Material). The HMM infers, for every trial, which is the more likely rewarding stimulus. Informative punishments are punishments that lead to a switch in behavior due to a switch in beliefs. Punishments that result in a behavioral switch without a belief switch are labeled uninformative as subjects appear to have acted erroneously with respect to their own belief. By defining events in this manner, it may be that the reward structure surrounding the events could differ between groups. This could confound the interpretation of any neural group differences. However, Supplementary Fig. S3 shows that reward rates before these events did not differ between groups (see Supplementary Data). The 22 healthy controls with good model fit showed significant activation for the contrast ‘informative punishment – informative reward’, which identifies ‘informative errors’ that putatively guide behavioral adaptation, in bilateral ventral striatum (R: t = 3.932, [17 8 − 3], p_FWE corrected for VS VOI_ = 0.002; L: t = 3.919, [− 16 11 − 5], p_FWE corrected for VS VOI_ = 0.002), the dmPFC (t = 7.641, [7 13 48], p_FWE whole brain_ < 0.001) and bilateral vlPFC (R: t = 7.038, [47 18 3], p_FWE whole brain_ < 0.001; L: t = 6.635, [− 30 23 − 5], p_FWE whole brain_ = 0.001).

Group comparison between HC and all SZ patients (including all patients, both with good and poor model fit, n = 22) showed that patients displayed reduced BOLD response in bilateral ventral striatum (R: t = 3.621; [17 8 − 5]; p_FWE corrected for VS VOI_ = 0.0052 and L: t = 3.733; [− 16 8 − 5]; p_FWE corrected for VS VOI_ = 0.004), as well as dmPFC (t = 4.260; [12 18 40]; p_FWE corrected for main effect_ = 0.003) and the right vlPFC (R: t = 4.070, [47 18 0], p_FWE corrected for main effect_ = 0.005). The group difference in the left vlPFC approached significance (L: t = 3.036, [− 33 20 − 10], p_FWE corrected for main effect_ = .092) (see also Supp. Table 3 and 4).

**Fig. 5:**
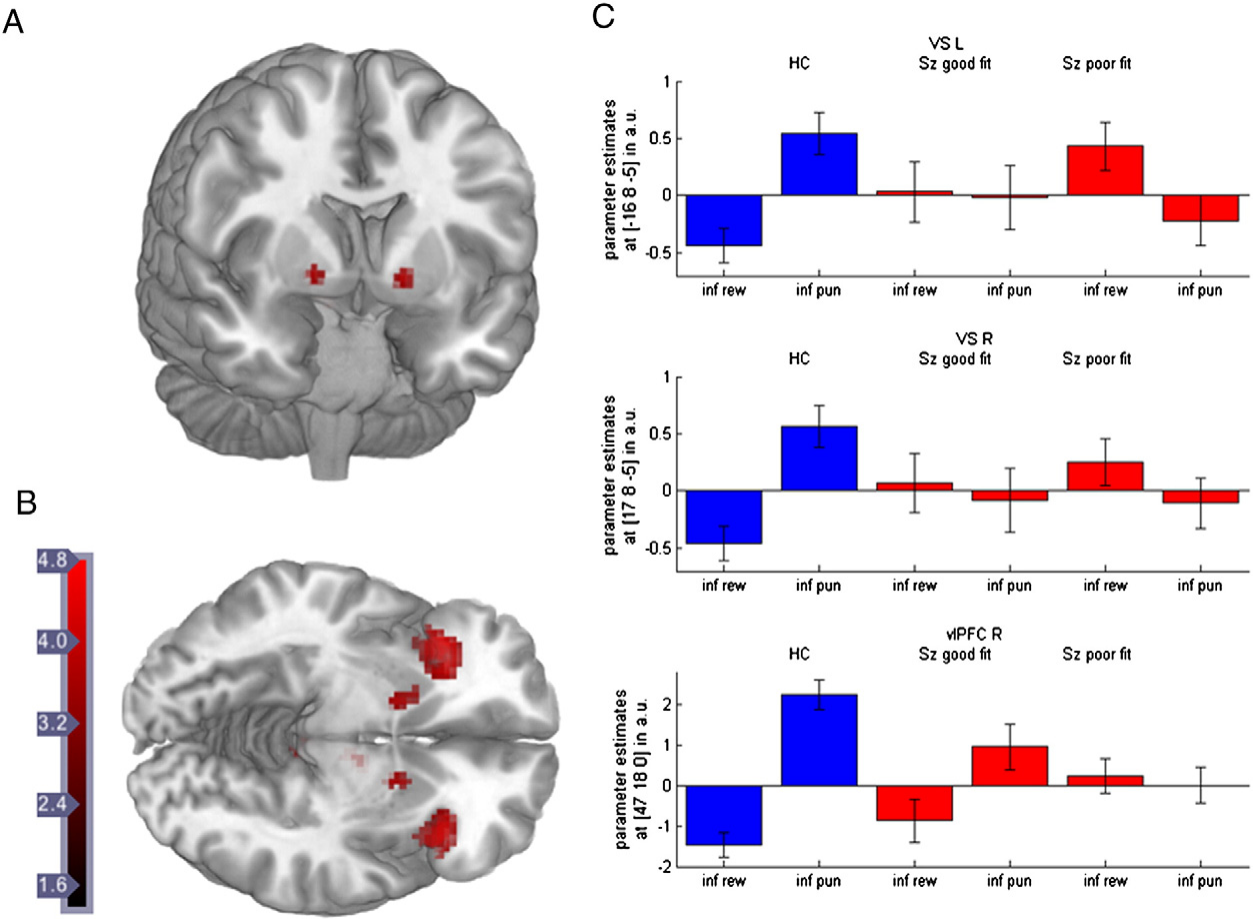
Group difference for the contrast ‘informative punishment – informative reward’. A + B: Healthy controls compared to schizophrenia patients (combining patients with good and bad model fit) displayed stronger activation in the bilateral ventral striatum (VS) and right ventrolateral prefrontal cortex (vlPFC) for the contrast ‘informative punishment – informative reward’ derived from the HMM model. C: Plots of parameter estimates revealed that patients with good and bad model fit showed reduced VS activation compared to healthy controls (upper and middle panel), while only patients with bad model fit showed reduced activation in the right vlPFC (lower panel) (for post-hoc t-tests — see results section).

Examining the parameter estimates of the subgroups indicated that reduced ventral striatal activation was present in SZ patients with good as well as with poor model fit, while reduced prefrontal activation was only observed for patients with poor model fit (see Fig. 5). Post-hoc t-tests showed that in right ventral striatum controls differed from both patient groups, while the two patient groups did not differ from each other (**VS R**: HC > poor-fit patients t = 3.51, [17 3 − 5]; p_FWE corrected for VS VOI_ = 0.008; HC > good-fit patients t = 2.81, [17 8 − 5]; p_FWE corrected for VS VOI_ = 0.045; good fit > poor fit patients p_FWE corrected for VS VOI_ > 0.1), while in the left ventral striatum good fit patients had only a trendwise reduced activation compared to controls and showed a trendwise higher activation compared to poor-fit patients (**VS L**: HC > poor-fit patients t = 4.02, [− 16 6 − 3], p_FWE corrected for VS VOI_ = 0.002; HC > good-fit patients t = 2.45, [− 16 8 − 5]; p_FWE corrected for VS VOI_ = 0.095; good-fit > poor-fit patients t = 2.52, [− 6 11 − 10]; p_FWE corrected for VS VOI_ = 0.083). In the prefrontal areas, good-fit patients differed from poor fit patients but not from healthy controls (**vlPFC R**: HC > poor fit patients t = 4.54, [47 18 0]; p_FWE corrected for main effect_ = 0.001; HC > good fit patients p_FWE corrected for main effect_ > 0.2; good-fit > poor-fit patients t = 3.45, [32 23 8]; p_FWE corrected for main effect_ = 0.030; **dmPFC**: HC > poor-fit patients t = 4.67, [10 16 45], p = 0.001; HC > good-fit patients p_FWE corrected for main effect_ > 0.2; good-fit > poor-fit patients t = 3.28, [10 13 45]; p_FWE corrected for main effect_ = 0.049).

### Comparison of drug-naïve vs. previously medicated patients

To address the effect of medication history in this sample of unmedicated schizophrenia patients, the subgroup of medication-free, but previously medicated patients (n = 9) was compared to the subgroup of drug-naïve patients (n = 15). On the behavioral level no differences were observed for the percentage of correct responses (drug-naïve: 65.4 +/− 8.53; medication-free: 62.4 +/− 5.56; p > .3), the number of learned conditions (drug-naïve: 5.27 +/− 1.75; medication-free: 4.22 +/− 1.64; p > .1) nor for the number of subjects with good model fit (10/15 drug-naïve and 3/9 medication-free patients with good HMM model fit; Chi-square test p > .1). On the neuronal level a trend for a higher fMRI PE activation in the left VS (t = 2.47, [− 11 13 − 5], p_FWE corrected for VS PE VOI_ = 0.089) was observed in the previously medicated patient group (n = 9) compared to the drug-naïve patients (n = 13). No difference was observed in the right VS (p_FWE corrected for VS PE VOI_ > .5) or outside the VS for the PE activation. The neuronal correlates of subjectively perceived reversal errors derived from the HMM showed no difference between drug-naïve and previously medicated patients (2-sample *t*-test for the contrast ‘informative punishment – informative reward’) neither in the VS (p_FWE corrected for VS VOI_ > .3) nor outside the VS.

## Discussion

To the best of our knowledge, this is the first observation of a neural signature of a reversal learning deficits in unmedicated schizophrenia patients. We show that ventral striatal dysfunction is a central element of this deficit in unmedicated patients. A key aspect of our results is that this difference persists and drives group differences even when we use detailed computational methods to discount the impact of possible different strategies employed by individual patients.

### Behavioral deficit during reversal learning

Behavioral results revealed, in line with previous findings, that patients performed worse than controls during reversal learning (Elliott et al., 1995; Leeson et al., 2009; Murray et al., 2008a; Pantelis et al., 1997; Waltz and Gold, 2007; Waltz et al., 2013). This was due to two distinct factors, namely a reinforcement insensitivity and a tendency to switch. First, low reinforcement sensitivity identified subjects who learned poorly. Poorly fitted patients also tended to have more severe positive PANSS scores. Reinforcement insensitivity likely generalizes broadly across tasks, and could contribute to an increased error rate across the entire sequence of tasks in the intradimensional/extradimensional set shift task sequence (Leeson et al., 2009). This observation is consistent with the broad inability of converting subjective utility into behavioral choice reported in severely affected schizophrenia patients (Gold et al., 2008; Heerey et al., 2008; Murray et al., 2008a). Second, modeling allows us to identify a potential underlying behavioral mechanism in schizophrenia patients more directly by asking whether, and to what extent, the use of a strategy actually predicts participants' behavior. This approach identified tendency to switch as a key factor, which was observed to be significantly increased in schizophrenia patients. Such an increased switch tendency may prima facie appear to be at odds with research on chronic schizophrenia (Elliott et al., 1995; Kerns and Berenbaum, 2002; Pantelis et al., 1997), which has emphasized perseverative features of choice. Our finding of an increased switch tendency is in line with recent research in first-episode psychosis. For example, in a probabilistic reversal task, Waltz and Gold (2007) report that schizophrenia patients complete fewer reversals. We see the same pattern where the increase in switching prevents subjects from reaching reversal criterion. In a more explicit, non-probabilistic, intradimensional/extradimensional set shift task (Murray et al., 2008a), patients showed significantly more errors at nearly every stage, which appears also be explicable by reinforcement insensitivity and/or an abnormally high switch tendency. However, due to the fact that subjects in our task do not have to reach a certain criterion before a reversal occurred, perseveration could not be tested specifically. Nevertheless, in line with the observed learning deficit here, a recent study of probabilistic category learning without a reversal component revealed reduced acquisition rates in schizophrenia patients compared to controls (Weickert et al., 2009). Another recent study applied computational modeling and found that schizophrenia patients, especially those with high negative symptoms, showed deficits in speeding up their reaction times to increase positive outcomes (Go learning) as well as deficits in the exploration of alternative actions (Strauss et al., 2011). In line with our finding, increased switching behavior was also observed during a probabilistic learning task with a reversal component in medicated, chronic schizophrenia patients (Waltz et al., 2013).

### Reduced prediction error signal in the ventral striatum

The analysis of neural prediction error correlates revealed ventral striatal hypofunction in unmedicated schizophrenia patients, in accordance with several previous studies assessing VS activation during processing of reward-indicating stimuli or PEs (Juckel et al., 2006b; Koch et al., 2010; Murray et al., 2008b; Schlagenhauf et al., 2009; Waltz et al., 2009). However, behavioral modeling revealed that a substantial number of patients were not fit better than chance even by the most parsimonious model, the HMM. When excluding these in relation to the fMRI analysis of prediction errors, reduced ventral striatal activation was still present trendwise in the reduced sample.

Our result here is in line with reduced striatal activation during appetitive and aversive Pavlovian conditioning in schizophrenia (Jensen et al., 2008; Waltz et al., 2009). It is also mirrored by similar relative VS hypoactivation in unmedicated patients when anticipating rewards in a monetary incentive delay task (Juckel et al., 2006a; Schlagenhauf et al., 2009), and reduced VS correlates of reward prediction errors in instrumental conditioning (Gradin et al., 2011; Morris et al., 2011; Murray et al., 2008b; Nielsen et al., 2012b but see; Romaniuk et al., 2010). Thus, ventral striatal dysfunction appears to contribute to the impairment in reversal learning in a manner that is consistent with its impairment in related aspects of reinforcement learning; it may primarily reflect motivational aspects of reinforcement learning (Robinson and Berridge, 2008). Moreover, this impairment was not secondary to antipsychotic medication, which can impair reward processing in schizophrenia (Heinz, 2002; Juckel et al., 2006a,b; Kapur, 2003; Schlagenhauf et al., 2008). The trendwise higher prediction error signal in the left VS in previously medicated patients compared to drug-naïve patients might indicate an ameliorating effect of previous antipsychotic medication on VS PE signaling deficits. Although due to limited sample size (n = 9 previously medicated vs. n = 15 drug-naïve patients) and the merely trend wise significance this has to be treated with caution and replicated in an independent larger sample.

### Subgroups according to task strategy

It appears important to compare patients and controls that perform a task at a similar level according to task strategy as applied in previous studies of cognitive deficits in schizophrenia (Callicott et al., 2003; Tan et al., 2006). With regard to reinforcement learning, this aim can be reached by using modeling techniques that incorporate the task structure e.g. the mirror symmetry between good and bad option in our reversal learning task. Consequently, we extended the findings from Rescorla–Wagner learning algorithms to computational models like HMM, which allowed us to assess the extent to which subjects were able to infer and use the latent structure of the task. The comparison of the different models fitted to the data (for more details see supplementary material) rests on Bayesian model comparison, and we additionally generated simulation data from the model to ensure that it is able to fully capture the patterns observed. Thus, rather than just testing aspects of our hypotheses by looking at specific parts of the data, we tested the behavioral predictions in their entirety (see (Huys et al., 2011) for a more detailed discussion). Based on this, behavior in patients and controls was best described by a more complex model of cognitive mapping, a Hidden Markov model.

The application of the HMM model revealed a subgroup of schizophrenia patients with a good model fit who used a similar strategy compared to healthy controls and another subgroup with a poor model who did not use the task structure. This allowed us to assess compensatory processes in schizophrenia patients who are able to solve the task. As demonstrated in the present study, this helps to reveal behavioral deficits and neurobiological impairments that are not confounded by differences in strategies between patients and controls (Price and Friston, 1999).

Using an fMRI analysis based on ‘subjective reversal errors’ identified by the HMM, healthy controls activated a striatal-cortical network that was previously reported to be involved in behavioral adaption (Cools et al., 2002). Group comparison demonstrated reduced activation in patients' ventral striatum, vlPFC and dmPFC. When directly comparing the three subgroups of the sample (controls, patients fit by the HMM and patients not fit by the HMM), we demonstrate that VS activation was reduced in both patient groups compared to controls separately. Remarkably, good fit patients did not differ from controls in terms of activation in the vlPFC and dmPFC and did show a higher activation in the same region compared to the poorly fit patients. Poorly fit patients – characterized by more severe psychotic symptoms – did not recruit prefrontal areas and showed less behavioral adaptation. The finding that more severe psychotic patients were less able to infer the task structure might be related to their inability to recruit prefrontal areas and instead relay on less complex strategies as indicated by the model comparison in this subgroup. This is in line with previous research on cognitive deficits in schizophrenia which demonstrate compensatory effects of prefrontal activation when patients were able to perform the task (Deserno et al., 2012; Tan et al., 2006). In contrast, the finding of reduced striatal activation in both patient sub-groups indicates that VS dysfunction contributes to reversal learning deficits in schizophrenia patients even when controlling for differences in task strategy, as implemented here using detailed computational modeling. This may point to a common biological alteration even in a heterogeneous population of schizophrenia patients.

Theoretical work (Heinz, 2002; Kapur, 2003), and previous work in our group (Schlagenhauf et al., 2013), support the idea that VS hypo-activation in schizophrenia may be related to elevated levels of dopamine. Indeed, prediction error BOLD correlates are inversely correlated with dopamine synthesis capacity (measured by FDOPA PET) in the ventral striatum (Schlagenhauf et al., 2012). This indicates that more ‘noisy’ neural prediction error signals may be a consequence of elevated dopamine levels, or through a reduced inhibition of phasic release via presynaptic dopamine D_2_ receptors (e.g. Bello et al., 2011). Elevated dopamine synthesis capacity as measured with FDOPA PET is among the best replicated imaging findings in schizophrenia, as recently highlighted in a meta-analyses (Fusar-Poli and Meyer-Lindenberg, 2013; Howes et al., 2012). It appears conceivable that altered striatal dopamine transmission, and its relation to hypothetical glutamatergic dysfunction are important features and potential common characteristics of the pathophysiology of schizophrenia patients as proposed in the framework of a ‘dysconnectivity hypothesis’ (Stephan et al., 2009).

## Conclusion

Taken together, a computational approach to reinforcement learning with a reversal component is applicable in human studies and animal experiments, and thus represents an important translational tool (Ragland et al., 2009). The combined neuroimaging and computational modeling approach adopted here, revealed deficits in reversal learning in unmedicated schizophrenia patients, which manifest as a decreased sensitivity to reward and as dysfunctional switching. These went along with decreased activation in the ventral striatum, a key area of the so-called dopaminergic reward system. Extending the approach to include multimodal imaging with PET and fMRI (Fusar-Poli et al., 2010; Kienast et al., 2008) will be important in clarifying the complex interaction of dopamine and glutamate in such learning impairments in schizophrenia.

## Financial disclosures

Dr. Schlagenhauf reports having received lecture fees from BMS and received funding from the German Research Foundation (SCHL 1969/1-1&2-1) and by the Max Planck Society. Dr. Huys, Dr. Beck and Mr. Deserno reported no biomedical financial interests or potential conflicts of interest. Dr. Rapp reports lecture fees from Glaxo Smith Kline, Servier, and Johnson & Johnson. Dr. Heinz has received research funding from the German Research Foundation (HE 2597/4-3; 7-3; 13-1;14-1; FOR 16/17; Excellence Cluster Exc 257 & STE 1430/2-1) and the German Federal Ministry of Education and Research (01GQ0411; 01QG87164; NGFN Plus 01 GS 08152 and 01 GS 08 159). This work was supported by the Wellcome Trust [Ray Dolan Senior Investigator Award 098362/Z/12/Z]. The Wellcome Trust Centre for Neuroimaging is supported by core funding from the Wellcome Trust 091593/Z/10/Z.

## Figures and Tables

**Table 1:** Group description.

	Schizophrenia patients	Healthy controls	Sig.
Age (years)	27.5 ± 5.2 (21–40)	27.2 ± 4.9 (20–41)	n.s.
Gender	22 males, 2 females	22 males, 2 females	
Edinburgh handedness inventory	84.6 ± 27.2 (n = 23)	90.7 ± 17.6	n.s.
Verbal IQ (WST)	97.7 ± 12.6	103.6 ± 8.6 (n = 23)	n.s.
Age of onset (years)	25.4 ± 5.8		
Duration of illness (years)	2.4 ± 2.2		
Number of episodes	1.7 ± 1.1		
PANSS total	85.6 ± 16.2		
PANSS positive	22.2 ± 5.8		
PANSS negative	21.6 ± 5.9		
PANSS general	41.8 ± 10.0		
