# Supplementary material for: Striatal dysfunction during reversal learning in unmedicated schizophrenia patients
Source: Neuroimage. 2014 Apr 1;89(100):171–80. doi: 10.1016/j.neuroimage.2013.11.034 (PMC3991847; doi:10.1016/j.neuroimage.2013.11.034)
Supplement: Supplementary file 1 — Supplementary material. [file mmc1.zip › nim10989-mmc1.docm]

Striatal dysfunction during reversal learning in unmedicated schizophrenia patients

**Florian Schlagenhauf, MD, MA^1,2*^, Quentin J.M. Huys, MA, MBBS, PhD^3*^, Lorenz Deserno, MD^1,2^, Michael A. Rapp, MD, PhD^1,4^, Anne Beck, PhD^1^, Hans-Joachim Heinze, MD^5,6^, Ray Dolan, MD^7,8^,**

**Andreas Heinz, MD^1,9^**

^1^ Department of Psychiatry and Psychotherapy, Campus Charité Mitte, Charité - Universitätsmedizin Berlin, Germany; ^2^Max Planck Institute for Human Cognitive and Brain Sciences, Leipzig, Germany; ^3^Gatsby Computational Neuroscience Unit and Wellcome Trust Centre for Neuroimaging, University College London, London WC1N 3BG, UK; and Translational Neuromodeling Unit, Department of Biomedical Engineering, ETH Zurich and University of Zurich; and Department of Psychiatry, Psychotherapy and Psychosomatics, University Hospital of Psychiatry Zurich, Zurich, Switzerland;^4^Department of Psychiatry, Mount Sinai School of Medicine, New York, NY; ^5^*Leibniz Institute for Neurobiology, Otto-von-Guericke University, Magdeburg, Germany; ^6^ Department of Neurology, Otto-von-Guericke University, Magdeburg, Germany; ^7^* Wellcome Trust Centre for Neuroimaging, University College London, London WC1N 3BG, UK; *^8^* *Humboldt-Universität zu Berlin School of Mind and Brain, Berlin, Germany;* ^9^ Cluster of Excellence NeuroCure, Charité-Universitätsmedizin Berlin, Berlin, Germany

### Corresponding author:

Florian Schlagenhauf, MD

Department of Psychiatry and Psychotherapy

Charité – Universitätsmedizin Berlin, Campus Mitte

Charitéplatz 1, 10117 Berlin, Germany

Tel. +49-30-450 517289

Fax. +49-30-450 517944

E-mail: [florian.schlagenhauf@charite.de](mailto:florian.schlagenhauf@charite.de)

# Supplementary Methods

## fMRI acquisition

Functional imaging was conducted using a 3 Tesla GE Signa scanner with an 8 channel phased array head coil to acquire gradient echo T2*-weighted echo-planar images. Per session, 310 EPI volumes (12 min) containing 29 slices (4 mm thick) were acquired with the following parameters: repetition time (TR) 2.3 s, echo time (TE) 27 ms, matrix size 128x128 and a field of view (FOV) of 256 x 256 mm², thus yielding an in-plane voxel resolution of 2 x 2 mm², flip =90 degrees. A T1 weighted structural data were collected for anatomical localization (TR=7.8 ms, TE=3.2 ms, matrix size 256x256, 1 mm slice thickness, voxel size of 1 mm³, flip =20 degree).

## fMRI data analyses

One patient’s imaging data was corrupted due to technical malfunction of storage device. Functional imaging data were analyzed using SPM8 (Wellcome Department of Imaging Neuroscience, Institute of Neurology, London, UK; http://www.fil.ion.ucl.ac.uk/spm/software/spm8/). ArtRepair was used to remove noise spikes and to repair bad slices within a particular scan and bad slices were repaired by interpolation between adjacent slices (“Noise Filtering”, <http://cibsr.stanford.edu/tools/ArtRepair/ArtRepair.htm>). The first three volumes of each session were discarded to allow for magnetic saturation effects. Images were corrected for delay in slice time acquisition and for motion using a rigid body transformation. Computing the Euklidic distance for the translation movement parameters revealed no significant group difference between schizophrenia patients and controls (t=1.469, p=.149). The individual anatomical T1 image was coregistered to the individual mean EPI image and then segmented using the SPM8 algorithms. Spatial normalization into Montreal Neurological Institute (MNI) space was performed by applying the normalization parameters derived during the segmentation step to all EPI images which resulted in a voxel size of 2.5 x 2.5 x 2.5 mm³. Spatial smoothing was applied with 8mm full width half maximum kernel.

Ventral striatal volume of interest

Small volume correction was used within the VS volume of interest (VOI). The VS VOI was constructed based on coordinates of previous findings using an in house tool provided by Torsten Wüstenberg similar to (Schlagenhauf et al., 2012) to create an fMRI-literature based probabilistic VOI for the VS. To this end, we selected 16 recent papers containing data from 325 subjects (Bray and O'Doherty, 2007; Cohen, 2007; Cohen and Ranganath, 2005; D'Ardenne et al., 2008; Gershman et al., 2009; Kahnt et al., 2009; Krugel et al., 2009; Murray et al., 2008; O'Doherty et al., 2004; O'Doherty et al., 2003; Palminteri et al., 2009; Pessiglione et al., 2006; Rodriguez et al., 2006; Schonberg et al., 2010; Tobler et al., 2006; Valentin and O'Doherty, 2009). From each study, the coordinates of PE related activation for right and the left VS were extracted (see (Schlagenhauf et al., 2012) for further details).

## Reinforcement learning models

Three classes of models were fitted: a stimulus-action Rescorla-Wagner model (SA), a double-update stimulus-action model (DSA), and a Hidden Markov Model (HMM).

Our reinforcement learning models assigned each action *a_t_* a probability based on an action value *Q(a_t_)* according to a softmax:

The models differed in terms of how this action value was constructed. For the stimulus-action Rescorla-Wagner model (SA), this was

$$Q_{t}\left( a_{t} \right)= Q_{t-1}\left( a_{t} \right)+ \varepsilon(\rho r_{t}-Q_{t-1}(a_{t}))$$

where $\varepsilon$ was the learning rate. Reinforcements entered the equation through $r_{t}\in\left\{ -1,1 \right\}$ and $\rho$ was a free parameter that determined the effective size of reinforcements for every subject. Model SA R/P allowed this last parameter to take on two different values depending on whether a reward or a punishment was received.

The double-update model (DSA) also used equation 1 to map $Q$ values to action probabilities. In this model, however, all $Q$ values for all actions were updated on every trial:

$$Q_{t}\left( a_{t} \right)= Q_{t-1}\left( a_{t} \right)+ \varepsilon(\rho r_{t}-Q_{t-1}(a_{t}))$$

$$Q_{t}\left( \bar{a}_{t} \right)= Q_{t-1}\left( \bar{a}_{t} \right)+ \varepsilon(-\rho r_{t}-Q_{t-1}(\bar{a}_{t}))$$

Thus, every time a reward was obtained for action $a$, the $Q$ value for its action was increased, while that of the other action $\bar{a}$ was reduced. The double update model DSA R/P again allowed for the parameter $\rho$ to take on two different values, one for rewards and one for punishments.

The Hidden Markov Model (HMM) models the hypothesis that subjects choose their action based on their belief $b(s)$ about the underlying state of the task $s$. The crux in formulating a model based on this, is to assume that in inferring this belief distribution over states, subjects neglect their policy, i.e. they treat action-reward pairs as simple observations $o_{t}=\{a_{t}, r_{t}\}$. The belief variable then takes on a role similar to the $Q$ value in the standard reinforcement learning models above, in the sense that is not really a hidden variable that is being averaged over for action choice, but rather a deterministic function of the past observations and the parameters.

Let the belief over states at time $t+1$, based on all observations (action-reward pairs) up to time $t$be $p\left( s_{t+1} | o^{t} \right)=p(s_{t+1}|a^{t},r^{t})$, where $o^{t}={\{o_{\tau}\}}_{\tau=1}^{t}= {\{a_{\tau},r_{\tau}\}}_{\tau=1}^{t}$. We write:

$$p\left( s_{t+1}|o^{t} \right)= \int ds_{t} p\left( s_{t+1} | s_{t} \right)\frac{p\left( o_{t}|s_{t} \right)p\left( s_{t}|o^{t-1} \right)}{\int d{s'}_{t} p\left( o_{t} | s_{t} \right)p(s_{t}|o^{t-1})}$$

The probability of an observation is given by its 'compatibility' with the state. A reward tells us with probability $c$ that we chose correctly and that the state is the state corresponding to action $a$. In model HMM R/P, a punishment tells us with probability $d$ that the state is the one not corresponding to action $a$.

where$0\leq c, d\leq1$. For clarity, an alternative way of expressing this is:

Note that in the model HMM (as opposed to HMM R/P), rewards and punishments are treated as equally informative, i.e. $c=d$.

Although the task really contains three states, subjects do not know this. Hence, we assume that subjects only care about which of the two actions is best, i.e. that there are only two states corresponding to each action being the better one. The probability of staying in a state is $\gamma$, and hence:

$$p\left( s_{t+1} | s_{t} \right)= \left\{ \begin{aligned} \gamma if s_{t+1}= s_{t} \\ 1-\gamma if s_{t+1}\neq s_{t} \end{aligned} \right.$$

Finally, the current belief $b=p(s_{t}=1|o^{t-1} )$ about the state of the task based on past observations is mapped into action probabilities via a sigmoidal function as in the reinforcement learning models, except that the steepness is fixed to 20:

Note that we do not infer the steepness of the sigmoid $\beta$ as this trades off with the state estimate.

For the HMM analysis we inferred the probability over states for each time-step. See Supplementary Results below for the definition of informative events.

### Model fitting and comparison

Some of these models are more flexible than others, and hence should a priori fit any data better. To account for this, we compute the ΔBIC score, which approximates the group-level log Bayes factors ratio. This assesses model quality (parsimony) by properly trading off model complexity against the tightness of the model fit (Huys et al., 2011).

# Supplementary Behavioral Results

### Model comparison

Three classes of models were fitted: a stimulus-action Rescorla-Wagner model (SA), a double-update stimulus-action model (DSA), and a Hidden Markov Model (HMM). Each of these could be augmented by an extra parameter allowing rewards and punishments to have differential impacts (R/P). Figure 3A shows that the most parsimonious model (model HMM R/P) was a Hidden Markov Model with separate reward and punishment sensitivities. This model maintained a probability distribution over the possible states of the task, where each state corresponds to one of the two stimuli being the correct one to choose. The model had three parameters: two captured participants’ sensitivity to rewards and punishments; a further parameter captured their beliefs about how likely the task state was to remain the same on each trial.

These model comparison results provided three key insights: First, participants treated rewards and punishments differently, as augmenting any model with separate reward and punishment sensitivities (models R/P) improved parsimony. Second, participants understood the mirror symmetry inherent in the task, whereby a punishment for the currently chosen stimulus indicates that the other stimulus might have been rewarded. This is shown by the fact that double-update models (DSA), in which a reward for action 1 is treated as an equal sized loss for action 2 (and vice versa), outperforms a standard reinforcement learning model in which participants iteratively learn two separate values for the two actions available (models SA). Third, the fact that the HMM R/P model outperformed model DSA R/P is important: in the former model, changes in the hidden states are step-wise, real transitions, while the DSA model implicitly assumes a gradual drift in the expected rewards for each stimulus-action pair. I.e. the HMM models can model the fact that reversals are sudden, whereas the other models assume that the reversals are gradual. Participants were thus differentially sensitive to rewards and punishments; and they grasped the two central aspects of the reinforcement schedule i.e. anti-correlated stimulus values and sudden transitions.

Supplementary Figure S1 shows that the same pattern as in Figure 3A nevertheless still holds when only considering subjects fitted better than chance.


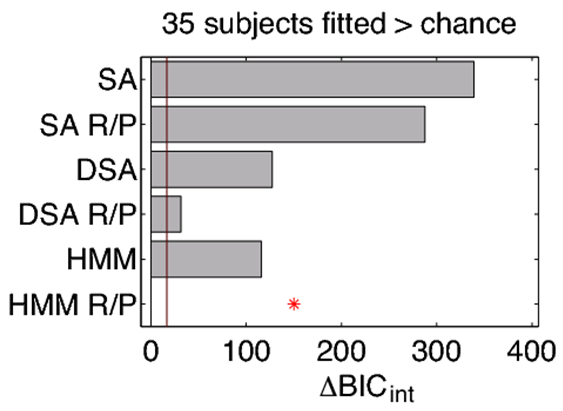


### Supplementary Figure S1:

Differential model Bayesian Information Criterion (ΔBIC_int_) scores (compared to the best model) for participants fitted better than chance. Asterisks indicate the best fitting model.

The red lines in Figure 3 A,D and S1 indicate the random-effects, individual-level, thresholds for ΔBIC_int_ differences. This shows that model HMM R/P provides a significant advantage in parsimony over all other models both at group and individual level.

As a further test, we generated surrogate data by running the HMM R/P model on the precise task the participants were presented with. This showed, that parametric variation in the HMM R/P model was able to replicate the learning curves of all three groups in great detail (dashed lines in Figure 3C). The same was true for the very first trials before the first reversal (data not shown).

## Informative and uninformative feedback

Reversal tasks demand a subtle balance between neglecting feedback that is misleading, but reacting to feedback that is informative. Standard event-based analyses (Cools et al., 2012) rely on a definition of misleading and informative that is only available to the experimenter. For instance, the final reversal errors are the final punished trials before a subject decides to switch, when the state of the task has truly switched. These trials are relevant because they define the key structure of the task. However, they are not visible to the subjects, but must be inferred by them. Instead of using on knowledge only available to the experimenter, the HMM can be used to define such key events using knowledge available to each individual subject and based on inferences that account for the actual behavior.

Using the HMM R/P model, we inferred subjects’ beliefs over the current state of the experiment for each time-point. An informative reward was defined as one that occurred after a choice that was consistent with the participant’s belief about the state of the experiment, and that was followed by a ’stay’, i.e. the same action. A reward was also an informative reward if the model indicated, for instance, that subjects believed the left side to be correct, but erroneously chose the right, still received a reward and then switched to the right. Similarly, an informative punishment was one that occurred after a choice consistent with the subjects’ belief and followed by a switch in their belief about the current state of the task. This thus defines informative events as seen by the subject, rather than as imposed by the experimenter.

Supplementary Figure S2 shows that controls and well-fit patients treated feedback differently. A three-way ANOVA with factors Reward/Punishment, Control/Patient and True/Belief revealed a main effect of Reward/Punishment (F(1,132)= 96.97, p<.001). Critically, there was an interaction of Reward/Punishment and True/Belief factors (F(1,132)= 15.2, p<0.001), showing that all subjects (controls and patients) treated rewards as more, and punishments as less informative than they were in reality (i.e. than the experimenter knew they were; panel A). It also revealed an interaction between the factors Reward/Punishment and Control/Patient (F(1,132)= 11.78, p<0.001). Finally, the difference in how informative rewards and punishments were believed to be was more pronounced in controls than in patients (F(1,66)=5.04, p=0.028; panel B).


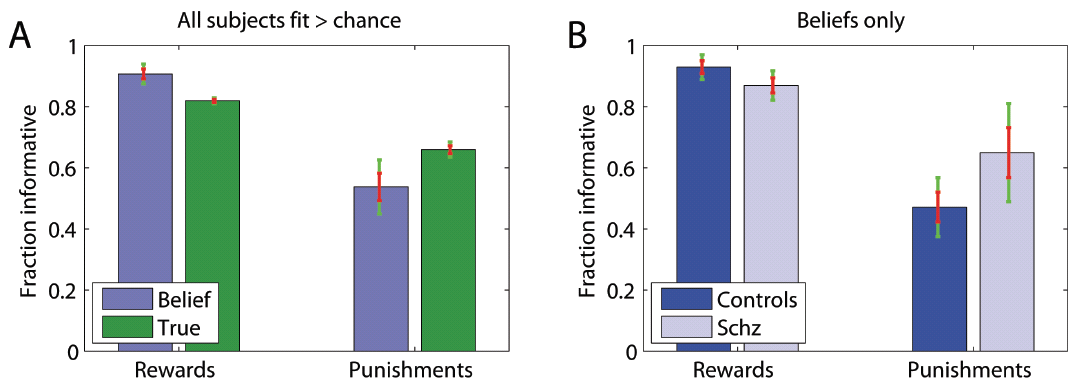


**Supplementary Figure S2:** Informative and uninformative feedback in patients and controls that are well fit. Both plots show the fraction of reinforcements of one type (rewards or punishments) that were truly informative, or that were believed to be informative by subjects according to model HMM (R/P).A: Overall, participants treat rewards as more, and punishments as less informative than they really are. Blue bars show the fraction of rewards and punishments treated as informative. Green bars show the fraction that is truly informative. B: The difference in how informative rewards and punishments are is more marked in controls than in patients.

Finally, it is worth emphasizing that the informative punishments will be used to identify times in the experiment when patients and controls perform as similar a cognitive operation as possible. The interpretation of any neural differences between the groups would be confounded if the informative punishments had a different reinforcement surrounding in the two groups. Figure S3 shows the average number of rewards preceding informative punishments in all three groups and reveals that this was not the case.

**Supplementary Figure S3:** Groups do not differ in terms of average reward probability prior to reversals or informative punishments. Each line shows the empirically estimated probability of reward prior to an informative punishment as defined above. The three groups are shown in different colors. Error bars represent standard errors of the mean.

# Supplementary fMRI Results

### Supplementary Table S1:

Whole brain results for the prediction error related activity of each group separately at the lenient threshold of p<0.01 uncorrected and p<0.05 FDR-corrected at the cluster level.

| **Region** | **Cluster extend** | **P _FDR cluster_** | **t-value** | **P _FWE voxel_** | **x** | **y** | **z** |
| --- | --- | --- | --- | --- | --- | --- | --- |
|  |  |  |  |  |  |  |  |
| 24 healthy controls |  |  |  |  |  |  |  |
| Superior Frontal Gyrus | 570 | 0.022 | 7.496 | 0.004 | 2 | 26 | 48 |
| Medial Frontal Gyrus | |  | 4.706 | 0.570 | -1 | 33 | 38 |
| Insula | 201 | 0.179 | 6.331 | 0.037 | 32 | 23 | -3 |
| Supramarginal Gyrus | 1012 | 0.002 | 4.901 | 0.441 | 57 | -50 | 35 |
| Inferior Parietal Lobule | |  | 4.469 | 0.731 | 47 | -50 | 55 |
| Angular Gyrus |  |  | 4.208 | 0.880 | 42 | -60 | 35 |
| Inferior Parietal Lobule | |  | 4.139 | 0.910 | 50 | -42 | 53 |
| Superior Parietal Lobule | |  | 3.548 | 0.999 | 37 | -72 | 43 |
| Precentral Gyrus | 494 | 0.026 | 4.805 | 0.503 | 35 | 21 | 38 |
| Middle Frontal Gyrus | |  | 4.099 | 0.925 | 50 | 16 | 40 |
| Lentiform Nucleus | 452 | 0.027 | 4.623 | 0.626 | 20 | 3 | 0 |
| Caudate |  |  | 4.387 | 0.783 | 15 | 11 | 8 |
| Midbrain |  |  | 4.147 | 0.906 | 7 | -12 | -13 |
| Inferior Parietal Lobule | 294 | 0.086 | 3.946 | 0.966 | -48 | -60 | 48 |
|  |  |  |  |  |  |  |  |
| 22 schizophrenia patients | |  |  |  |  |  |  |
| Superior Parietal Lobule | 725 | 0.013 | 5.164 | 0.342 | -31 | -70 | 45 |
| Superior Parietal Lobule | |  | 5.089 | 0.381 | -33 | -60 | 50 |
| Precuneus |  |  | 4.854 | 0.518 | -13 | -70 | 45 |

### Supplementary Table S2:

Whole brain results for the prediction error related activity of the group differences between healthy controls (HC) and schizophrenia patients (Sz) with good and poor model fit at the lenient threshold of p<0.001 uncorrected with a cluster extend of more than 10 voxels. Please note that no cluster survived correction for multiple comparisons (also not using an initial uncorrected p-value of p<0.01).

| **Region** | **Cluster extend** | **P _FDR cluster_** | **t-value** | **P _FWE voxel_** | **x** | **y** | **z** |
| --- | --- | --- | --- | --- | --- | --- | --- |
|  |  |  |  |  |  |  |  |
| 24 healthy controls > 22 schizophrenia patients | | | | | |  |  |
| Midbrain | 20 | 0.296 | 3.764 | 0.000 | 5 | -25 | -23 |
| Midbrain |  |  | 3.381 | 0.001 | -6 | -22 | -20 |
| Inferior Frontal Gyrus | 23 | 0.296 | 3.744 | 0.000 | 30 | 13 | -10 |
|  |  |  |  |  |  |  |  |
| 22 schizophrenia patients > 24 healthy controls | | | | | |  |  |
| Superior Parietal Lobule | 44 | 0.824 | 4.154 | 0.000 | -33 | -60 | 50 |
| Claustrum | 46 | 0.824 | 3.949 | 0.000 | -33 | -7 | 8 |
| Superior Temporal Gyrus |  |  | 3.638 | 0.000 | -48 | -10 | 3 |
| Precuneus | 11 | 0.824 | 3.482 | 0.001 | -16 | -82 | 43 |

### Supplementary Table S3:

Whole brain results for the contrast ‘informative punishment - informative reward’ of each group separately at the lenient threshold of p<0.01 uncorrected and p<0.05 FDR-corrected at the cluster level.

| **Region** | **Cluster extend** | **P _FDR cluster_** | **t-value** | **P _FWE voxel_** | **x** | **y** | **z** |
| --- | --- | --- | --- | --- | --- | --- | --- |
|  |  |  |  |  |  |  |  |
| 22 healthy controls with good model fit | | | | | |  |  |
| Superior Frontal Gyrus | 2616 | 0.000 | 7.640 | 0.000 | 7 | 13 | 48 |
| Cingulate Gyrus |  |  | 5.478 | 0.004 | -8 | 23 | 35 |
| Middle Frontal Gyrus |  |  | 4.954 | 0.027 | 35 | 3 | 45 |
| Superior Frontal Gyrus |  |  | 4.504 | 0.111 | 12 | 6 | 63 |
| Superior Frontal Gyrus |  |  | 4.107 | 0.324 | -11 | 1 | 65 |
| Inferior Frontal Gyrus | 1495 | 0.001 | 7.038 | 0.000 | 47 | 18 | 3 |
| Lentiform Nucleus |  |  | 3.931 | 0.476 | 17 | 8 | -3 |
| Caudate |  |  | 3.190 | 0.984 | 15 | 6 | 13 |
| Inferior Frontal Gyrus | 1235 | 0.002 | 6.635 | 0.000 | -31 | 23 | -5 |
| Insula |  |  | 6.454 | 0.000 | -38 | 21 | 0 |
| Lentiform Nucleus |  |  | 4.073 | 0.351 | -16 | 13 | -5 |
| Thalamus |  |  | 3.336 | 0.949 | -11 | -7 | 3 |
| Caudate |  |  | 2.834 | 1.000 | -11 | 1 | 8 |
| Middle Frontal Gyrus | 405 | 0.062 | 4.433 | 0.137 | 37 | 31 | 25 |
| Cerebellum | 440 | 0.059 | 4.378 | 0.160 | 5 | -32 | -20 |
| Inferior Parietal Lobule | 629 | 0.025 | 4.224 | 0.242 | 40 | -47 | 38 |
| Supramarginal Gyrus |  |  | 4.084 | 0.342 | 52 | -45 | 28 |
| Inferior Parietal Lobule |  |  | 3.665 | 0.734 | 37 | -62 | 38 |
|  |  |  |  |  |  |  |  |
| 13 schizophrenia patients with good fit | | |  |  |  |  |  |
| Superior Frontal Gyrus | 3715 | 0.000 | 6.076 | 0.000 | -8 | 3 | 65 |
| Medial Frontal Gyrus |  |  | 5.044 | 0.020 | 10 | 13 | 45 |
| Precentral Gyrus |  |  | 4.771 | 0.049 | -51 | -2 | 45 |
| Middle Frontal Gyrus |  |  | 3.787 | 0.617 | 32 | -7 | 63 |
| Superior Frontal Gyrus |  |  | 3.785 | 0.619 | 5 | 23 | 55 |
| Cingulate Gyrus |  |  | 3.678 | 0.721 | -6 | 21 | 30 |
| Superior Frontal Gyrus |  |  | 3.641 | 0.755 | 40 | 41 | 28 |
| Middle Frontal Gyrus |  |  | 3.486 | 0.875 | 55 | 26 | 30 |
| Angular Gyrus | 881 | 0.031 | 4.567 | 0.092 | 35 | -57 | 35 |
| Superior Parietal Lobule |  |  | 4.101 | 0.329 | 37 | -70 | 45 |
| Precuneus |  |  | 3.497 | 0.868 | 20 | -70 | 53 |
| Supramarginal Gyrus |  |  | 3.139 | 0.990 | 55 | -47 | 23 |
| Inferior Parietal Lobule | 754 | 0.039 | 4.184 | 0.268 | -51 | -47 | 40 |
| Precuneus |  |  | 3.914 | 0.492 | -11 | -62 | 45 |
| Superior Temporal Gyrus |  |  | 3.817 | 0.587 | -33 | -55 | 30 |
|  |  |  |  |  |  |  |  |
| 9 schizophrenia patients with poor model fit | | | |  |  |  |  |
| n.s. |  |  |  |  |  |  |  |
|  |  |  |  |  |  |  |  |

### Supplementary Table S4:

Whole brain results for the contrast ‘informative punishment - informative reward’ of the group differences between healthy controls (HC) and schizophrenia patients (Sz) with good and poor model fit at the lenient threshold of p<0.01 uncorrected and p<0.05 FDR-corrected at the cluster level.

| **Region** | **Cluster extend** | **P _FDR cluster_** | **t-value** | **P _FWE voxel_** | **x** | **y** | **z** |
| --- | --- | --- | --- | --- | --- | --- | --- |
|  |  |  |  |  |  |  |  |
| 22 HC with good model fit > 13 Sz patients with good model fit | | | | |  |  |  |
| n.s. |  |  |  |  |  |  |  |
|  |  |  |  |  |  |  |  |
| 13 Sz with good model fit > 22 HC with good model fit | | | | |  |  |  |
| Inferior Temporal Gyrus | 1316 | 0.009 | 5.010 | 0.022 | -58 | -55 | -5 |
| Superior Temporal Gyrus |  |  | 4.012 | 0.402 | -46 | -52 | 18 |
| Lingual Gyrus |  |  | 3.606 | 0.786 | -31 | -57 | -3 |
| Middle Temporal Gyrus |  |  | 3.339 | 0.948 | -51 | -35 | -3 |
| Transverse Temporal Gyrus |  |  | 3.029 | 0.997 | -61 | -10 | 10 |
| Parahippocampal Gyrus |  |  | 2.991 | 0.998 | -28 | -50 | 5 |
| Insula |  |  | 2.946 | 0.999 | -38 | -45 | 13 |
| Sub-Gyral |  |  | 2.926 | 0.999 | -28 | -42 | 0 |
| Superior Temporal Gyrus |  |  | 2.824 | 1.000 | -66 | -27 | 5 |
| Middle Temporal Gyrus |  |  | 2.730 | 1.000 | -61 | -35 | 3 |
| Middle Occipital Gyrus |  |  | 2.629 | 1.000 | -46 | -67 | 3 |
| Precentral Gyrus |  |  | 2.508 | 1.000 | -48 | -5 | 10 |
| Caudate |  |  | 2.471 | 1.000 | -31 | -42 | 13 |
|  |  |  |  |  |  |  |  |
| 22 HC with good model fit > 9 Sz patients with poor model fit | | | | | |  |  |
| Inferior Frontal Gyrus | 4356 | 0.000 | 5.023 | 0.021 | 47 | 18 | 5 |
| Insula |  |  | 4.998 | 0.023 | -38 | 18 | 3 |
| Culmen |  |  | 4.894 | 0.033 | -1 | -32 | -20 |
| Medial Frontal Gyrus |  |  | 4.672 | 0.067 | 10 | 16 | 45 |
| Lentiform Nucleus |  |  | 4.019 | 0.396 | -16 | 6 | -3 |
| Cingulate Gyrus |  |  | 3.687 | 0.713 | -8 | 23 | 38 |
| Superior Frontal Gyrus |  |  | 3.663 | 0.735 | -6 | 13 | 48 |
| Medial Frontal Gyrus |  |  | 3.546 | 0.833 | -8 | 18 | 45 |
| Lentiform Nucleus |  |  | 3.506 | 0.862 | 17 | 3 | -5 |
| Inferior Frontal Gyrus |  |  | 3.326 | 0.953 | 62 | 18 | 20 |
| Superior Temporal Gyrus |  |  | 3.323 | 0.954 | -36 | 6 | -20 |
| Thalamus |  |  | 3.266 | 0.970 | -11 | -10 | 3 |
| Caudate |  |  | 3.262 | 0.971 | -6 | 6 | 15 |
| Caudate |  |  | 3.230 | 0.978 | 7 | 13 | 5 |
|  |  |  |  |  |  |  |  |
| 9 Sz patients with poor model fit > 22 HC with good model fit | | | | |  |  |  |
| n.s. | | | | |  |  |  |
|  | | | | |  |  |  |
| 13 Sz patients with good model fit > 9 Sz patients with model poor fit | | | | | | |  |
| n.s. | | | | | | |  |
|  | | | | | | |  |
| 9 Sz patients with poor model fit > 13 Sz patients with good model fit | | | | | | |  |
| n.s. |  |  |  |  |  |  |  |
|  |  |  |  |  |  |  |  |

Reference List

Bray, S., O'Doherty, J., 2007. Neural coding of reward-prediction error signals during classical conditioning with attractive faces. Journal of Neurophysiology 97, 3036-3045.

Cohen, M.X., 2007. Individual differences and the neural representations of reward expectation and reward prediction error. Social Cognitive and Affective Neuroscience 2, 20-30.

Cohen, M.X., Ranganath, C., 2005. Behavioral and neural predictors of upcoming decisions. Cognitive Affective & Behavioral Neuroscience 5, 117-126.

D'Ardenne, K., McClure, S.M., Nystrom, L.E., Cohen, J.D., 2008. BOLD responses reflecting dopaminergic signals in the human ventral tegmental area. Science 319, 1264-1267.

Gershman, S.J., Pesaran, B., Daw, N.D., 2009. Human Reinforcement Learning Subdivides Structured Action Spaces by Learning Effector-Specific Values. Journal of Neuroscience 29, 13524-13531.

Huys, Q.J., Cools, R., Golzer, M., Friedel, E., Heinz, A., Dolan, R.J., Dayan, P., 2011. Disentangling the roles of approach, activation and valence in instrumental and pavlovian responding. PLoS Comput.Biol. 7, e1002028.

Kahnt, T., Park, S.Q., Cohen, M.X., Beck, A., Heinz, A., Wrase, J., 2009. Dorsal striatal-midbrain connectivity in humans predicts how reinforcements are used to guide decisions. J.Cogn Neurosci. 21, 1332-1345.

Krugel, L.K., Biele, G., Mohr, P.N.C., Li, S.C., Heekeren, H.R., 2009. Genetic variation in dopaminergic neuromodulation influences the ability to rapidly and flexibly adapt decisions. Proceedings of the National Academy of Sciences of the United States of America 106, 17951-17956.

Murray, G.K., Corlett, P.R., Clark, L., Pessiglione, M., Blackwell, A.D., Honey, G., Jones, P.B., Bullmore, E.T., Robbins, T.W., Fletcher, P.C., 2008. Substantia nigra/ventral tegmental reward prediction error disruption in psychosis. Mol.Psychiatry 13, 239, 267-239, 276.

O'Doherty, J., Dayan, P., Schultz, J., Deichmann, R., Friston, K., Dolan, R.J., 2004. Dissociable roles of ventral and dorsal striatum in instrumental conditioning. Science 304, 452-454.

O'Doherty, J.P., Dayan, P., Friston, K., Critchley, H., Dolan, R.J., 2003. Temporal difference models and reward-related learning in the human brain. Neuron 38, 329-337.

Palminteri, S., Boraud, T., Lafargue, G., Dubois, B., Pessiglione, M., 2009. Brain Hemispheres Selectively Track the Expected Value of Contralateral Options. Journal of Neuroscience 29, 13465-13472.

Pessiglione, M., Seymour, B., Flandin, G., Dolan, R.J., Frith, C.D., 2006. Dopamine-dependent prediction errors underpin reward-seeking behaviour in humans. Nature 442, 1042-1045.

Rodriguez, P.F., Aron, A.R., Poldrack, R.A., 2006. Ventral-striatal/nucleus-accumbens sensitivity to prediction errors during classification learning. Human Brain Mapping 27, 306-313.

Schlagenhauf, F., Rapp, M.A., Huys, Q.J., Beck, A., Wustenberg, T., Deserno, L., Buchholz, H.G., Kalbitzer, J., Buchert, R., Bauer, M., Kienast, T., Cumming, P., Plotkin, M., Kumakura, Y., Grace, A.A., Dolan, R.J., Heinz, A., 2012. Ventral striatal prediction error signaling is associated with dopamine synthesis capacity and fluid intelligence. Hum.Brain Mapp.

Schonberg, T., O'Doherty, J.P., Joel, D., Inzelberg, R., Segev, Y., Daw, N.D., 2010. Selective impairment of prediction error signaling in human dorsolateral but not ventral striatum in Parkinson's disease patients: evidence from a model-based fMRI study. Neuroimage 49, 772-781.

Tobler, P.N., O'Doherty, J.P., Dolan, R.J., Schultz, W., 2006. Human neural learning depends on reward prediction errors in the blocking paradigm. Journal of Neurophysiology 95, 301-310.

Valentin, V.V., O'Doherty, J.P., 2009. Overlapping Prediction Errors in Dorsal Striatum During Instrumental Learning With Juice and Money Reward in the Human Brain. Journal of Neurophysiology 102, 3384-3391.
